# *trans*-Bis(acetato-κ*O*)diaquabis­(2-amino­pyrazine-κ*N* ^4^)manganese(II) dihydrate

**DOI:** 10.1107/S1600536811028583

**Published:** 2011-07-23

**Authors:** Shan Gao, Seik Weng Ng

**Affiliations:** a Key Laboratory of Functional Inorganic Material Chemistry, Ministry of Education, Heilongjiang University, Harbin 150080, People’s Republic of China; b Department of Chemistry, University of Malaya, 50603 Kuala Lumpur, Malaysia, and, Chemistry Department, Faculty of Science, King Abdulaziz University, PO Box 80203 Jeddah, Saudi Arabia

## Abstract

The Mn^II^ atom in the title compound, [Mn(CH_3_COO)_2_(C_4_H_5_N_3_)_2_(H_2_O)_2_]·2H_2_O, is situated on a center of inversion and shows an octa­hedral coordination polyhedron made up by four O atoms and two N atoms. The octa­hedron is somewhat tetra­gonally distorted owing to the longer Mn—N bond [2.323 (3) Å]. The mononuclear complex mol­ecule and uncoordinated water mol­ecules are linked by O—H⋯N, N—H⋯O and O—H⋯O hydrogen bonds, generating a three-dimensional network.

## Related literature

For the crystal structure of manganese acetate dihydrate, see: Cheng & Wang (1991 ▶).
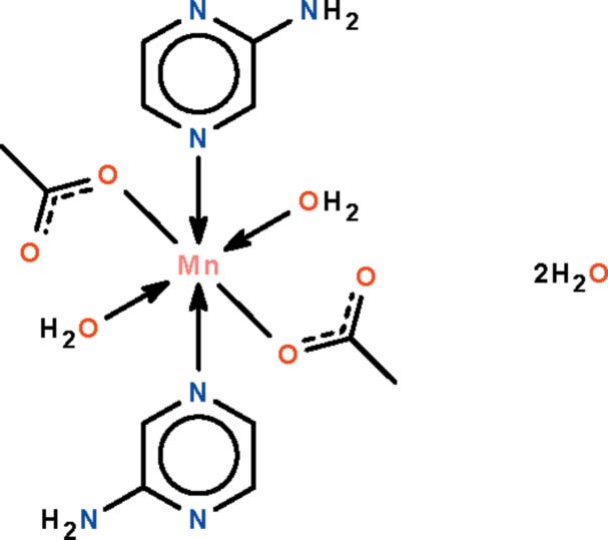


## Experimental

### 

#### Crystal data

[Mn(C_2_H_3_O_2_)_2_(C_4_H_5_N_3_)_2_(H_2_O)_2_]·2H_2_O
*M* *_r_* = 435.31
Triclinic, 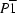

*a* = 7.0761 (7) Å
*b* = 8.5411 (8) Å
*c* = 9.5162 (10) Å
α = 100.866 (3)°
β = 105.036 (3)°
γ = 110.250 (3)°
*V* = 495.92 (9) Å^3^
*Z* = 1
Mo *K*α radiation
μ = 0.72 mm^−1^
*T* = 293 K
0.10 × 0.08 × 0.05 mm

#### Data collection

Rigaku R-AXIS RAPID IP diffractometer
Absorption correction: multi-scan (*ABSCOR*; Higashi, 1995 ▶) *T*_min_ = 0.932, *T*_max_ = 0.965
4911 measured reflections
2249 independent reflections
1558 reflections with *I* > 2σ(*I*)
*R*_int_ = 0.033

#### Refinement

*R*[*F* ^2^ > 2σ(*F* ^2^)] = 0.043
*wR*(*F* ^2^) = 0.159
*S* = 1.07
2249 reflections
148 parameters
8 restraints
H atoms treated by a mixture of independent and constrained refinement
Δρ_max_ = 0.80 e Å^−3^
Δρ_min_ = −1.01 e Å^−3^

### 

Data collection: *RAPID-AUTO* (Rigaku, 1998 ▶); cell refinement: *RAPID-AUTO*; data reduction: *CrystalClear* (Rigaku/MSC, 2002 ▶); program(s) used to solve structure: *SHELXS97* (Sheldrick, 2008 ▶); program(s) used to refine structure: *SHELXL97* (Sheldrick, 2008 ▶); molecular graphics: *X-SEED* (Barbour, 2001 ▶); software used to prepare material for publication: *publCIF* (Westrip, 2010 ▶).

## Supplementary Material

Crystal structure: contains datablock(s) global, I. DOI: 10.1107/S1600536811028583/im2304sup1.cif

Structure factors: contains datablock(s) I. DOI: 10.1107/S1600536811028583/im2304Isup2.hkl

Additional supplementary materials:  crystallographic information; 3D view; checkCIF report

## Figures and Tables

**Table 1:** Hydrogen-bond geometry (Å, °)

*D*—H⋯*A*	*D*—H	H⋯*A*	*D*⋯*A*	*D*—H⋯*A*
O1w—H11⋯O2	0.84 (1)	1.89 (2)	2.690 (4)	160 (5)
O1w—H12⋯N2^i^	0.84 (1)	2.02 (2)	2.837 (4)	165 (5)
O2w—H21⋯O1^ii^	0.84 (1)	2.02 (1)	2.851 (4)	171 (4)
O2w—H22⋯O2^iii^	0.84 (1)	1.90 (2)	2.726 (5)	167 (5)
N3—H31⋯O2w	0.88 (1)	1.98 (1)	2.859 (5)	178 (6)

Symmetry codes: (i) 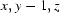; (ii) 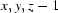; (iii) 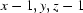.

**Fig. 1.**
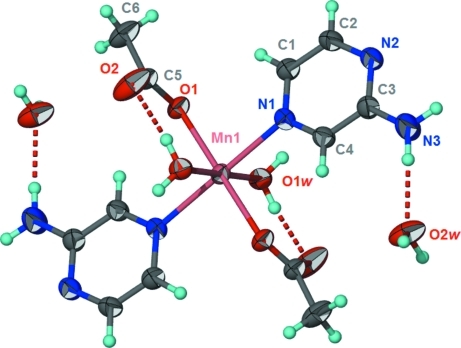
Thermal ellipsoid plot (Barbour, 2001) of Mn(H2O)2(C2H3O2)2(C4H5N3)2 × 2 H2O at the 50% probability level. Hydrogen atoms are drawn as spheres of arbitrary radius.

**Fig. 2.**
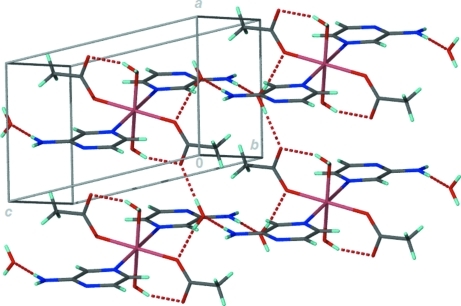
Three-dimensional hydrogen-bonded network of the title compound. Hydrogen bonds are depicted as dashed lines.

## supplementary crystallographic information

### Comment

There are few crystal structure studies of *N*-heterocyclic adducts of manganese acetate, the latter crystallizing as a dihydrate (Cheng & Wang, 1991). Other first-row transition metal acetates furnish a large number of adducts. The Mn^II^ atom in Mn(H_2_O)_2_(C_2_H_3_O_2_)_2_(C_4_H_5_N_3_)_2_ × 2 H_2_O (Scheme I, Fig. 1) shows an octahedral coordination polyhedron made up by four O atoms and two N atoms. The octahedron is somewhat tetragonally distorted owing to the longer Mn–N bond. The mononuclear complex molecule and lattice water molecules are linked hydrogen bonds to generate a three-dimensional network (Table 1, Fig. 2).

### Experimental

To an aqueous solution of 2-aminopyrazine (1 mmol) was added manganese acetate tetrahydrate (1 mmol). The mixture was stirred for 30 min and then filtered. Colorless crystals of the title complex separated from the solution after a few days.

### Refinement

Carbon-bound H-atoms were placed in calculated positions (C–H 0.93 Å) and were included in the refinement using the riding model approximation, with *U*(H) set to 1.2*U*(C). The amino and water H-atoms were located in a difference Fourier map, and were refined with distance restraints N–H 0.88±0.01 Å, O–H 0.84±0.01 Å and H···H 1.37±0.01 Å; their temperature factors were refined.

The largest peaks/holes in the final difference Fourier map were found in close vicinity of Mn1.

### Crystal data

**Table:**

[Mn(C_2_H_3_O_2_)_2_(C_4_H_5_N_3_)_2_(H_2_O)_2_]·2H_2_O	*Z* = 1
*M**_r_* = 435.31	*F*(000) = 227
Triclinic, *P*1	*D*_x_ = 1.458 Mg m^−^^3^
Hall symbol: -P 1	Mo *K*α radiation, λ = 0.71073 Å
*a* = 7.0761 (7) Å	Cell parameters from 3626 reflections
*b* = 8.5411 (8) Å	θ = 3.3–27.5°
*c* = 9.5162 (10) Å	µ = 0.72 mm^−^^1^
α = 100.866 (3)°	*T* = 293 K
β = 105.036 (3)°	Prism, colorless
γ = 110.250 (3)°	0.10 × 0.08 × 0.05 mm
*V* = 495.92 (9) Å^3^	

### Data collection

**Table:**

Rigaku R-AXIS RAPID IP diffractometer	2249 independent reflections
Radiation source: fine-focus sealed tube	1558 reflections with *I* > 2σ(*I*)
graphite	*R*_int_ = 0.033
ω scans	θ_max_ = 27.5°, θ_min_ = 3.3°
Absorption correction: multi-scan (*ABSCOR*; Higashi, 1995)	*h* = −8→9
*T*_min_ = 0.932, *T*_max_ = 0.965	*k* = −11→11
4911 measured reflections	*l* = −12→12

### Refinement

**Table:**

Refinement on *F*^2^	Secondary atom site location: difference Fourier map
Least-squares matrix: full	Hydrogen site location: inferred from neighbouring sites
*R*[*F*^2^ > 2σ(*F*^2^)] = 0.043	H atoms treated by a mixture of independent and constrained refinement
*wR*(*F*^2^) = 0.159	*w* = 1/[σ^2^(*F*_o_^2^) + (0.0633*P*)^2^ + 0.7384*P*] where *P* = (*F*_o_^2^ + 2*F*_c_^2^)/3
*S* = 1.07	(Δ/σ)_max_ = 0.001
2249 reflections	Δρ_max_ = 0.80 e Å^−^^3^
148 parameters	Δρ_min_ = −1.01 e Å^−^^3^
8 restraints	Extinction correction: *SHELXL97* (Sheldrick, 2008), Fc^*^=kFc[1+0.001xFc^2^λ^3^/sin(2θ)]^-1/4^
Primary atom site location: structure-invariant direct methods	Extinction coefficient: 0.021 (8)

### Fractional atomic coordinates and isotropic or equivalent isotropic displacement parameters (Å^2^)

**Table:**

	*x*	*y*	*z*	*U*_iso_*/*U*_eq_	
Mn1	0.5000	0.5000	0.5000	0.0358 (3)	
O1	0.6700 (4)	0.6113 (3)	0.7465 (3)	0.0413 (6)	
O2	0.9808 (5)	0.5959 (6)	0.7684 (4)	0.0826 (12)	
O1W	0.7647 (4)	0.4463 (3)	0.4659 (3)	0.0414 (6)	
H11	0.858 (6)	0.503 (5)	0.553 (3)	0.082 (18)*	
H12	0.763 (8)	0.346 (3)	0.440 (5)	0.09 (2)*	
O2W	0.4054 (5)	0.6764 (5)	−0.0904 (4)	0.0719 (10)	
H21	0.470 (6)	0.649 (7)	−0.147 (4)	0.085 (18)*	
H22	0.273 (2)	0.636 (6)	−0.141 (4)	0.084 (18)*	
N1	0.6498 (5)	0.7758 (4)	0.4722 (3)	0.0406 (7)	
N2	0.7601 (5)	1.1077 (4)	0.4338 (4)	0.0464 (8)	
N3	0.6301 (9)	0.9802 (5)	0.1732 (4)	0.0738 (13)	
H31	0.562 (9)	0.888 (5)	0.091 (4)	0.111*	
H32	0.654 (10)	1.085 (4)	0.163 (7)	0.111*	
C1	0.7478 (6)	0.9231 (5)	0.5927 (4)	0.0465 (9)	
H1	0.7803	0.9147	0.6914	0.056*	
C2	0.7999 (7)	1.0845 (5)	0.5717 (4)	0.0476 (9)	
H2	0.8665	1.1830	0.6576	0.057*	
C3	0.6692 (7)	0.9628 (5)	0.3141 (4)	0.0448 (9)	
C4	0.6154 (6)	0.7964 (5)	0.3351 (4)	0.0408 (8)	
H4	0.5537	0.6977	0.2497	0.049*	
C5	0.8583 (6)	0.6370 (5)	0.8229 (4)	0.0428 (8)	
C6	0.9380 (8)	0.7229 (7)	0.9942 (5)	0.0659 (13)	
H6A	1.0903	0.7569	1.0363	0.099*	
H6B	0.8658	0.6414	1.0402	0.099*	
H6C	0.9085	0.8248	1.0145	0.099*	

### Atomic displacement parameters (Å^2^)

**Table:**

	*U*^11^	*U*^22^	*U*^33^	*U*^12^	*U*^13^	*U*^23^
Mn1	0.0395 (5)	0.0376 (4)	0.0301 (4)	0.0194 (3)	0.0089 (3)	0.0084 (3)
O1	0.0417 (14)	0.0507 (15)	0.0297 (12)	0.0216 (12)	0.0082 (10)	0.0099 (11)
O2	0.0530 (19)	0.146 (3)	0.0437 (17)	0.054 (2)	0.0102 (14)	0.0016 (19)
O1W	0.0385 (14)	0.0421 (15)	0.0414 (15)	0.0189 (11)	0.0122 (12)	0.0067 (12)
O2W	0.0502 (19)	0.097 (3)	0.0474 (18)	0.0277 (19)	0.0103 (15)	−0.0094 (17)
N1	0.0447 (17)	0.0347 (15)	0.0428 (17)	0.0181 (13)	0.0142 (14)	0.0116 (13)
N2	0.057 (2)	0.0371 (16)	0.0435 (17)	0.0194 (14)	0.0172 (15)	0.0115 (14)
N3	0.124 (4)	0.048 (2)	0.042 (2)	0.030 (2)	0.022 (2)	0.0184 (17)
C1	0.054 (2)	0.045 (2)	0.0352 (19)	0.0192 (17)	0.0118 (16)	0.0099 (16)
C2	0.055 (2)	0.0359 (19)	0.041 (2)	0.0151 (17)	0.0117 (17)	0.0050 (16)
C3	0.055 (2)	0.042 (2)	0.0385 (19)	0.0226 (17)	0.0156 (17)	0.0115 (16)
C4	0.047 (2)	0.0354 (18)	0.0382 (19)	0.0155 (15)	0.0159 (16)	0.0093 (15)
C5	0.045 (2)	0.044 (2)	0.0321 (18)	0.0159 (16)	0.0069 (15)	0.0099 (15)
C6	0.059 (3)	0.094 (4)	0.033 (2)	0.034 (3)	0.0049 (19)	0.005 (2)

### Geometric parameters (Å, °)

**Table:**

Mn1—O1W^i^	2.163 (3)	N2—C3	1.335 (5)
Mn1—O1W	2.163 (3)	N2—C2	1.336 (5)
Mn1—O1^i^	2.181 (2)	N3—C3	1.344 (5)
Mn1—O1	2.181 (2)	N3—H31	0.879 (10)
Mn1—N1^i^	2.323 (3)	N3—H32	0.878 (10)
Mn1—N1	2.323 (3)	C1—C2	1.366 (5)
O1—C5	1.260 (4)	C1—H1	0.9300
O2—C5	1.232 (5)	C2—H2	0.9300
O1W—H11	0.840 (10)	C3—C4	1.406 (5)
O1W—H12	0.841 (10)	C4—H4	0.9300
O2W—H21	0.838 (10)	C5—C6	1.516 (5)
O2W—H22	0.838 (10)	C6—H6A	0.9600
N1—C4	1.321 (5)	C6—H6B	0.9600
N1—C1	1.348 (5)	C6—H6C	0.9600
			
O1W^i^—Mn1—O1W	180.000 (1)	C3—N3—H31	121 (4)
O1W^i^—Mn1—O1^i^	91.79 (10)	C3—N3—H32	119 (4)
O1W—Mn1—O1^i^	88.21 (9)	H31—N3—H32	119 (6)
O1W^i^—Mn1—O1	88.21 (10)	N1—C1—C2	120.9 (4)
O1W—Mn1—O1	91.79 (10)	N1—C1—H1	119.6
O1^i^—Mn1—O1	180.000 (1)	C2—C1—H1	119.6
O1W^i^—Mn1—N1^i^	90.34 (10)	N2—C2—C1	123.2 (3)
O1W—Mn1—N1^i^	89.66 (10)	N2—C2—H2	118.4
O1^i^—Mn1—N1^i^	89.93 (10)	C1—C2—H2	118.4
O1—Mn1—N1^i^	90.07 (10)	N2—C3—N3	118.3 (4)
O1W^i^—Mn1—N1	89.66 (10)	N2—C3—C4	120.7 (3)
O1W—Mn1—N1	90.34 (10)	N3—C3—C4	121.0 (3)
O1^i^—Mn1—N1	90.07 (10)	N1—C4—C3	122.1 (3)
O1—Mn1—N1	89.93 (10)	N1—C4—H4	118.9
N1^i^—Mn1—N1	180.000 (1)	C3—C4—H4	118.9
C5—O1—Mn1	128.8 (2)	O2—C5—O1	124.7 (3)
Mn1—O1W—H11	99 (3)	O2—C5—C6	118.0 (4)
Mn1—O1W—H12	125 (4)	O1—C5—C6	117.4 (4)
H11—O1W—H12	109 (2)	C5—C6—H6A	109.5
H21—O2W—H22	110 (2)	C5—C6—H6B	109.5
C4—N1—C1	116.8 (3)	H6A—C6—H6B	109.5
C4—N1—Mn1	120.7 (2)	C5—C6—H6C	109.5
C1—N1—Mn1	121.9 (2)	H6A—C6—H6C	109.5
C3—N2—C2	116.2 (3)	H6B—C6—H6C	109.5
			
O1W^i^—Mn1—O1—C5	−175.9 (3)	C4—N1—C1—C2	2.6 (6)
O1W—Mn1—O1—C5	4.1 (3)	Mn1—N1—C1—C2	−168.1 (3)
N1^i^—Mn1—O1—C5	93.7 (3)	C3—N2—C2—C1	−1.6 (6)
N1—Mn1—O1—C5	−86.3 (3)	N1—C1—C2—N2	−0.5 (6)
O1W^i^—Mn1—N1—C4	−95.9 (3)	C2—N2—C3—N3	−178.7 (4)
O1W—Mn1—N1—C4	84.1 (3)	C2—N2—C3—C4	1.5 (6)
O1^i^—Mn1—N1—C4	−4.2 (3)	C1—N1—C4—C3	−2.7 (5)
O1—Mn1—N1—C4	175.8 (3)	Mn1—N1—C4—C3	168.1 (3)
O1W^i^—Mn1—N1—C1	74.3 (3)	N2—C3—C4—N1	0.7 (6)
O1W—Mn1—N1—C1	−105.7 (3)	N3—C3—C4—N1	−179.1 (4)
O1^i^—Mn1—N1—C1	166.1 (3)	Mn1—O1—C5—O2	−2.8 (6)
O1—Mn1—N1—C1	−13.9 (3)	Mn1—O1—C5—C6	178.0 (3)

Symmetry codes: (i) −*x*+1, −*y*+1, −*z*+1.

### Hydrogen-bond geometry (Å, °)

**Table:**

*D*—H···*A*	*D*—H	H···*A*	*D*···*A*	*D*—H···*A*
O1w—H11···O2	0.84 (1)	1.89 (2)	2.690 (4)	160 (5)
O1w—H12···N2^ii^	0.84 (1)	2.02 (2)	2.837 (4)	165 (5)
O2w—H21···O1^iii^	0.84 (1)	2.02 (1)	2.851 (4)	171 (4)
O2w—H22···O2^iv^	0.84 (1)	1.90 (2)	2.726 (5)	167 (5)
N3—H31···O2w	0.88 (1)	1.98 (1)	2.859 (5)	178 (6)

Symmetry codes: (ii) *x*, *y*−1, *z*; (iii) *x*, *y*, *z*−1; (iv) *x*−1, *y*, *z*−1.
